# Advancing Biomarker Research: In Situ Cu Isotope Analysis in Liver Tumors by LA-MC-ICP-MS

**DOI:** 10.1021/acs.analchem.4c05626

**Published:** 2025-02-18

**Authors:** Mathias Schannor, Marcus Oelze, Heike Traub, Yubei He, Robin Schmidt, Luisa Heidemann, Lynn Jeanette Savic, Jochen Vogl, Björn Meermann

**Affiliations:** † Bundesanstalt für Materialforschung und -prüfung (BAM), Richard-Willstätter-Straße 11, 12489 Berlin, Germany; ‡ Department of Radiology, Charité - Universitätsmedizin Berlin Campus Virchow Klinikum (CVK), Augustenburger Platz 1, 13353 Berlin, Germany

## Abstract

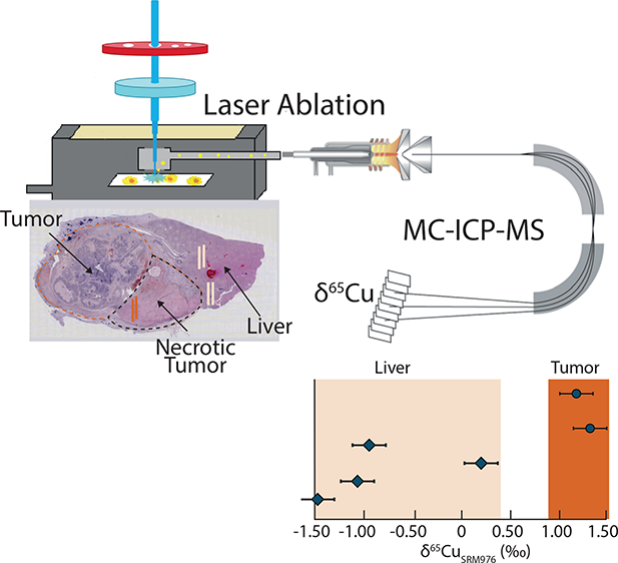

Stable metal isotopes have received increasing attention as medical biomarkers due to their potential to detect changes in metal metabolism related to diseases. In particular, copper stable isotopes are a powerful tool to identify isotopic variation between tumors and healthy tissue, suggesting application in cancer diagnosis. However, potential mechanisms causing isotope fractionation, such as redox- or bond-forming reactions and interactions of metals during transmembrane import and export, are less well understood. Here, we established an *in situ* method using laser ablation-multicollector-inductively coupled plasma-mass spectrometry (LA-MC-ICP-MS) to advance our understanding of the underlying processes responsible for isotope fractionation between normal and diseased tissues. Gelatin-based bracketing standards and quality control reference materials, crucial for laser ablation analysis, were developed to allow correction for instrumentally induced isotope fractionation during LA-MC-ICP-MS analysis. Using such matrix-matched standards, the method achieved intermediate precisions for delta values of better than 0.15 ‰ (2 *s*) for inorganic reference materials and of better than 0.17 ‰ (2 *s*) for biological reference materials. The developed routine was tested on rabbit VX2 liver tumor samples, a model system resembling human hepatocellular carcinoma (HCC) used to study liver cancer. *In situ* Cu isotope compositions between healthy ( = −1.5 ‰ to 0.2 ‰) and tumorous ( = 0.0 ‰ to 1.3 ‰) liver tissue show distinct differences in their isotope ratios. The observed isotopic dichotomy is consistent with previous solution-based MC-ICP-MS work, showing enrichment of heavy ^65^Cu in cancer biopsies relative to healthy tissue.

## Introduction

Natural isotopic compositions of essential mineral elements, such as Ca, Fe, Cu, or Zn, can be shifted in biological organisms as a reaction to metabolic changes or disruption of their cellular homeostasis.^1−3^ These physical processes and biochemical mechanisms involve reduction/oxidation or bond-forming reactions during metal uptake or release, which induce isotope fractionation. Such processes alter isotopic compositions because the lighter isotopes of a given element are more reactive than the heavier isotopes and are concentrated in reaction products, and the heavier isotopes prefer stronger bonds (e.g., when adsorbing to ligands).^4−6^ The induced variations have been measurable since the advent of multicollector-inductively coupled plasma-mass spectrometry (MC-ICP-MS) in the 1990s.^7−9^

Copper is a crucial nutrient for organisms since it is essential for mitochondrial respiration, enzyme activity, the maintenance of protein function, and Fe metabolism.^10−12^ Excessively low or high Cu concentrations can, however, severely disrupt physiological functions. For example, anomalous accumulations of Cu can cause oxidative stress, cellular damage, abnormal protein binding, and cytotoxicity.^13^ The two naturally occurring stable isotopes of Cu, ^63^Cu (69.2 %) and ^65^Cu (30.8 %) show measurable variation in their isotopic abundance in human serum in response to metabolic diseases.^14−17^ Different types of cancer enriched the lighter Cu isotope in the blood of patients relative to healthy controls.^17−19^ Bulk digestions of primary liver carcinoma (hepatocellular carcinoma) showed heavier Cu isotope compositions relative to healthy liver tissue,^16^ suggesting a link to the isotopically lighter blood serum, thus advocating Cu isotopes as a potential biomarker of cancer detection and for therapy monitoring. Possible explanations for the Cu isotope variations could be the cytosolic storage of isotopically heavy Cu chelated by lactate^17^ (Warburg effect), the release of intracellular Cu from cysteine clusters such as metallothionein,^16^ or lower reductase activity in the tumor.^20^

A drawback to this strong potential is the time-consuming sample preparation prior to isotopic analysis because the material needs to be dissolved using acids, and then Cu needs to be separated from the matrix by ion-exchange chromatography. *In situ* analysis by laser ablation MC-ICP-MS (LA-MC-ICP-MS) avoids tedious sample preparation and would enable direct measurements of tissue samples to detect isotopic variations between healthy and diseased cells. The physical processes and underlying mechanisms of isotope fractionation could be spatially resolved and quantified, helping to capitalize on the capability of metal isotopes as biomarkers. Nonetheless, LA-MC-ICP-MS analysis of biomedical samples is currently acutely limited due to the scarcity of matrix-matched reference materials. Often suitable reference materials cannot be produced for any application due to stability or complexity issues. However, such matrix-matched standards are necessary to correct for instrumental sources of isotope fractionation such as particle size distribution, ablation physics, and differential ionization.^21^ Recently, gelatin standards were established as matrix-matched standards in laser ablation bioimaging applications.^22−25^ Gelatin properties simulate the properties of a protein-rich cellular material, and their ionization efficiencies and particle transport behavior resemble that of a biological material.^25^

Here, we exploit these gelatin properties and developed matrix-matched Cu isotope standards for LA-MC-ICP-MS measurements of a biomedical sample material. The novel analytical routine is first tested by analyzing certified Cu reference materials admixed with gelatin. Secondly, the method is applied to VX2 tumor cells in rabbit livers, a model system for sarcoma mimicking biomedical imaging features of hepatocellular carcinoma,^26^ to show that differences in Cu isotope compositions can be spatially resolved between tumor and healthy liver tissues.

## Experimental Section

### Reagents and Materials

The primary Cu isotope reference material, NIST SRM 976, was used as the bracketing standard, while NIST SRM 3114 served as the secondary reference material to monitor the quality of isotope analysis. Both materials were obtained from the National Institute of Standards and Technology (NIST, USA). Biological reference materials included bovine liver SRM-1577c, purchased from NIST, and dogfish liver DOLT-5, acquired from the Canadian National Research Council (NRC). The candidate Cu isotope reference material BAM-i020 was also used for the performance control. Copper isotope values for these reference materials are listed in Table 1. Gelatin powder from cold water fish skin was purchased from Sigma-Aldrich. Superfrost soda-lime-silica glass microscope slides of 26 mm × 76 mm × 1 mm were acquired from Thermo Fisher Scientific.

**Table 1:** Comparison of the Recommended and Measured  Values for the Quality Control and/or Reference Materials Measured by LA-MC-ICP-MS

	/‰			
material	recommended	measured^e^	*U* (*k* = 2)	*N*^f^
NIST SRM 976^a^	0	0 ± 0.15		
NIST SRM 3114^b^	–0.0764 ± 0.0048	–0.03 ± 0.15	0.53	8
BAM-i020^b^	1.476 ± 0.004	1.45 ± 0.15	0.47	27
NIST SRM 1577c^c^	0.37 ± 0.05	0.39 ± 0.15	0.46	22
NRC DOLT-5^d^	–0.02 ± 0.08	–0.08 ± 0.17	0.54	12

a The exact value defining the  SRM 976 scale^31^ is shown.

b DSL mean of Ni-doped measurements with expanded uncertainty (*k* = 2) as obtained from CCQM-P213.^32^

c Mean value with reproducibility of 2 *s.*(33)

d Mean value with expanded uncertainty *U* with (*k* = 2).^34^

e Mean measured value with intermediate precision as 2 *s* is shown.

f Number of measurements.

### Digestion of Biological Reference Materials

Biological reference materials were digested to admix their pure solution with gelatin. Approximately 200 mg–400 mg of biological reference materials (NIST SRM 1577c and NRC DOLT-5) were weighed into 90 mL quartz glass digestion tubes, mixed with 3 mL concentrated HNO_3_ + 0.4 mL 30 % H_2_O_2_, and left for 2 h to react. The vessels were sealed with Teflon tape and placed into a high-pressure asher (HPA-S, Anton Paar, Graz, Austria) for 2 h of digestion at 240 °C at 100 bar. After digestion, the solutions were transferred to Savillex PFA screw top beakers and evaporated to dryness on a hot plate at 140 °C.

### Preparation of Gelatin Cu Isotope Standards

Fish skin gelatin stock solutions were prepared by weighing in gelatin powder and admixing Milli-Q water to make up a final gelatin mass fraction of 10%. The prepared gelatin solution was then homogenized on a thermomixer (Eppendorf ThermoMixerC, Hamburg, Germany) at 55 °C to obtain a clear solution. Copper reference material solutions were added to gelatin stock solutions to reach a final Cu mass fraction of 100 μg/g. Drops of 20 μL were deposited on heated glass microscope slides placed on a hot plate that was set at 120 °C producing dried gelatin droplets within seconds.

### VX2 Rabbit Liver Tumor Model

Animal experiments were approved and performed according to the animal protection committee of the Landesamt für Gesundheit and Soziales (LaGeSo), Berlin, Germany, the local Guidelines and Provisions for Implementation of the Animal Welfare Act by Charité - Universitätsmedizin Berlin, and the regulations of the Federation of Laboratory Animal Science Associations (FELASA).

The VX2 tumor is an epidermoid rabbit tumor induced by the *Shope papilloma* virus, which resembles the human hepatocellular carcinoma (HCC) from a biomedical imaging perspective.^26^ Hence, it is used as a model system to study liver cancer and cancer therapy.^27^ New Zealand white rabbits had VX2 samples inoculated into their hind limbs which served as the propagation of the tumor strain. Donor rabbits were anesthetized, and the VX2 tumors were explanted from the hind limbs. Subsequently, the donor VX2 tumor was injected into the left liver lobe of an anesthetized naive rabbit. Injected tumors were allowed to grow to diameters of approximately 1.5–2.0 cm.

Two different groups were analyzed: The control group, where rabbits carried liver tumors in situ and were not treated, and the microwave ablation group (MWA-group), where rabbits received ablation treatment described below. Tumor ablation is a nonsurgical approach for HCCs and other tumors that is minimally invasive and aims to obtain complete necrosis of the tumors. All ablations in this study refer to incomplete microwave ablation. Rabbits were anesthetized and the left lobe of the liver and the tumor were exposed. A microwave needle was inserted along the long axis of the tumor and frozen and the correct position was confirmed using computer tomography. Ablation was performed using a 17F microwave generator (power 30 W, duration 40 s, temperature 103 ± 3 °C around the microwave needle). The tumor was incompletely ablated to ensure that there was residual tumor after ablation. Thermal coagulation was performed along the needle tract at the end of the ablation to prevent bleeding from the needle tract. Double-layer suturing was performed when the needle was taken out.

Tumor tissues were extracted after the sacrifice of the animal, fixed in neutral buffered formalin with a formaldehyde mass fraction of 10 % in water, and paraffin-embedded. Paraffin blocks were sectioned into 5–10 μm thin slices and placed on Superfrost soda-lime-silica glass slides for laser ablation analysis. Additionally, cut slices were stained with hematoxylin and eosin (H&E).

### Instrumentation and Measurements

#### MC-ICP-MS

Copper-stable isotope ratio measurements were performed at BAM on a Neptune Plus MC-ICP-MS (Thermo Fisher Scientific, Bremen, Germany) for solution measurements as well as for laser ablation measurements. Tuning of gas flows, torch position, and ion optics of the MC-ICP-MS was conducted on a daily basis using a mixed Cu and Ni solution aspirated using an Apex Q (Elemental Scientific Inc., Omaha, USA) desolvating nebulizer to yield the highest stability and sensitivity. The ion optics of the MC-ICP-MS were operated at the medium and high mass resolution, with a typical mass resolution of m/Δ*m* > 6000 for high resolution, to resolve isobaric interferences in pseudohigh-resolution mode. Faraday detectors (equipped with 10^11^ Ohm amplifiers) were positioned to measure interference-free, flat-top peak shoulders on ^60^Ni (L3), ^61^Ni (L2), ^62^Ni (L1), ^63^Cu (C), and ^65^Cu (H3) isotopes.

Copper isotope values are reported in the delta notation according to Coplen^28^ as  relative to the international isotope measurement standard NIST SRM 976 in per mil (‰), following eq 1:
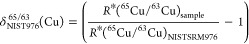
1

Details about the mass bias correction routine are given in the Supporting Information.

#### Laser Ablation

A NWR213 laser ablation system (213 nm, Elemental Scientific Laser, Bozeman, USA) equipped with a TwoVol1 ablation chamber was attached to the Neptune Plus MC-ICP-MS. Details on instrumental settings are summarized in Table S1. The laser system was controlled by ActiveView software (Elemental Scientific Laser, Bozeman, USA). Laser ablation in the ablation chamber was carried out in a helium atmosphere using a helium flow of 0.85 L min^–1^ and an additional argon flow of 0.9–1.0 L min^–1^ injected with a Y-connector before the torch of the MC-ICP-MS. The fluence was kept below the ablation threshold of glass to avoid ablation of the soda-lime-silica microscope slide, which can cause ^23^Na^40^Ar^+^ interferences on the ^63^Cu signal. Lines with a laser beam diameter of 110 μm were scanned across the sample surface for a duration of 100 s for each measurement. Analysis of samples or reference materials with a biological matrix was followed by a background measurement lasting 150 s to prevent subsequent memory effects.

The in-house-prepared gelatin material spiked with NIST SRM 976 as the Cu source served as the bracketing material. Furthermore, in each measurement session, a set of reference materials, such as DOLT-5 and NIST SRM 1577c, mixed with gelatin and deposited on glass slides, were regularly analyzed between sample measurements.

To process the raw isotope data and correct for background effects, we followed a previously published protocol.^29^ This protocol involved applying specific data acceptance and rejection criteria. Notably, only measured *r*(^65^Cu/^63^Cu) ratios deviating by less than 3 standard deviations from the sample mean (criterion ‘a’) were considered for calculation. Additionally, results with a mass bias drift of less than 0.30 ‰ between the two bracketing calibrators (criterion ‘b’) were accepted and reported in this study.

#### LA-ICP-ToF-MS Imaging

Multielemental imaging was performed to get insights into the distribution of Cu and other biometals within the samples and reference materials. A NWRimage laser ablation system (266 nm, Elemental Scientific Laser, Bozeman, USA) equipped with a low-dispersion ablation cell in a TwoVol3 ablation chamber was attached to an ICP-ToF-MS instrument (icpTOF 2R, TOFWERK AG, Thun, Switzerland) using a 1.016 mm ID PEEK tubing and an Ultrafast Dual Concentric Injector 2 (DCI2). The laser system was controlled by ActiveView2 software (Elemental Scientific Laser, Bozeman, USA). Details about the analytical setup, calibration methods, and data processing are given in the Supporting Information and Table S2.

## Results and Discussion

### Blank Contribution of Gelatin Droplets to LA-MC-ICP-MS Measurements

Several batches of gelatin droplet blanks with varying gelatin mass fractions (1 and 5% gelatin) were prepared to determine their Cu blank contribution to the measured Cu signal. The average ^63^Cu intensity of 5% gelatin blanks was within a range of 7–12 mV, whereas ^63^Cu intensities of 1% gelatin blanks were between 1 and 2 mV (Figure S1). Contributions of the high gelatin content blanks to the measured Cu signal of gelatin Cu standards are thus on average below 7%. In gelatin standards that were prepared with 1% gelatin, less than 1% of the entire Cu signal can be attributed to the gelatin. Such low contributions do not significantly affect the calculated  value and are, hence, negligible. Consequently, the gelatin reference materials were produced with 1% gelatin mass fractions.

### Isotopic Homogeneity and Stability of Gelatin Standards

**Figure 1:**
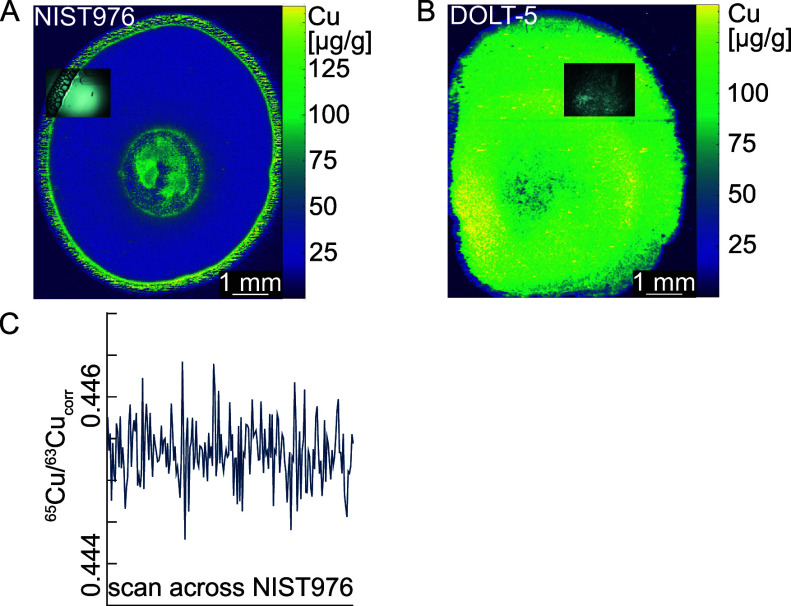
Copper elemental content images (LA-ICP-ToF-MS) for gelatin standard droplets of NIST SRM 976 and NRC DOLT-5. (A) NIST SRM 976 shows Cu enrichment in the rim and center of the droplet, and evenly distributed Cu contents in between. An inset microscopic image of the droplet displays an even surface. (B) NRC DOLT-5 (dogfish liver) reveals an overall uneven Cu concentration in the ToF image, and a patchy surface in the microscopic image. (C) Cross-sectional measurement (LA-MC-ICP-MS) across a NIST SRM 976 droplet indicates minimal variation in the measured *r*(^65^Cu/^63^Cu) ratio.

Elemental heterogeneity in dried gelatin droplet standards has been observed and can be attributed to kinetic effects, such as the donut-coffee-stain and Marangoni effects.^23,30^ Hence, potential isotope fractionation caused by kinetic effects during the drying of the gelatin droplets needs to be assessed. Therefore, elemental maps for the gelatin droplets were constructed using LA-ICP-ToF-MS. The ToF-image of a NIST SRM976 gelatin droplet shows enrichment of Cu within the rim and the central part of the gelatin droplet, consistent with the donut-coffee-stain and Marangoni effects (Figure 1A). Apart from the enrichment in the rim, Cu is homogeneously distributed within the droplet, also visible in the inset of the microscope picture (Figure 1A). A microscopic image of an NRC DOLT-5 droplet shows a “patchy” structure of the gelatin droplet, which is further corroborated by the heterogeneous Cu distribution within the gelatin, as revealed by the ToF image (Figure 1B).

**Figure 2:**
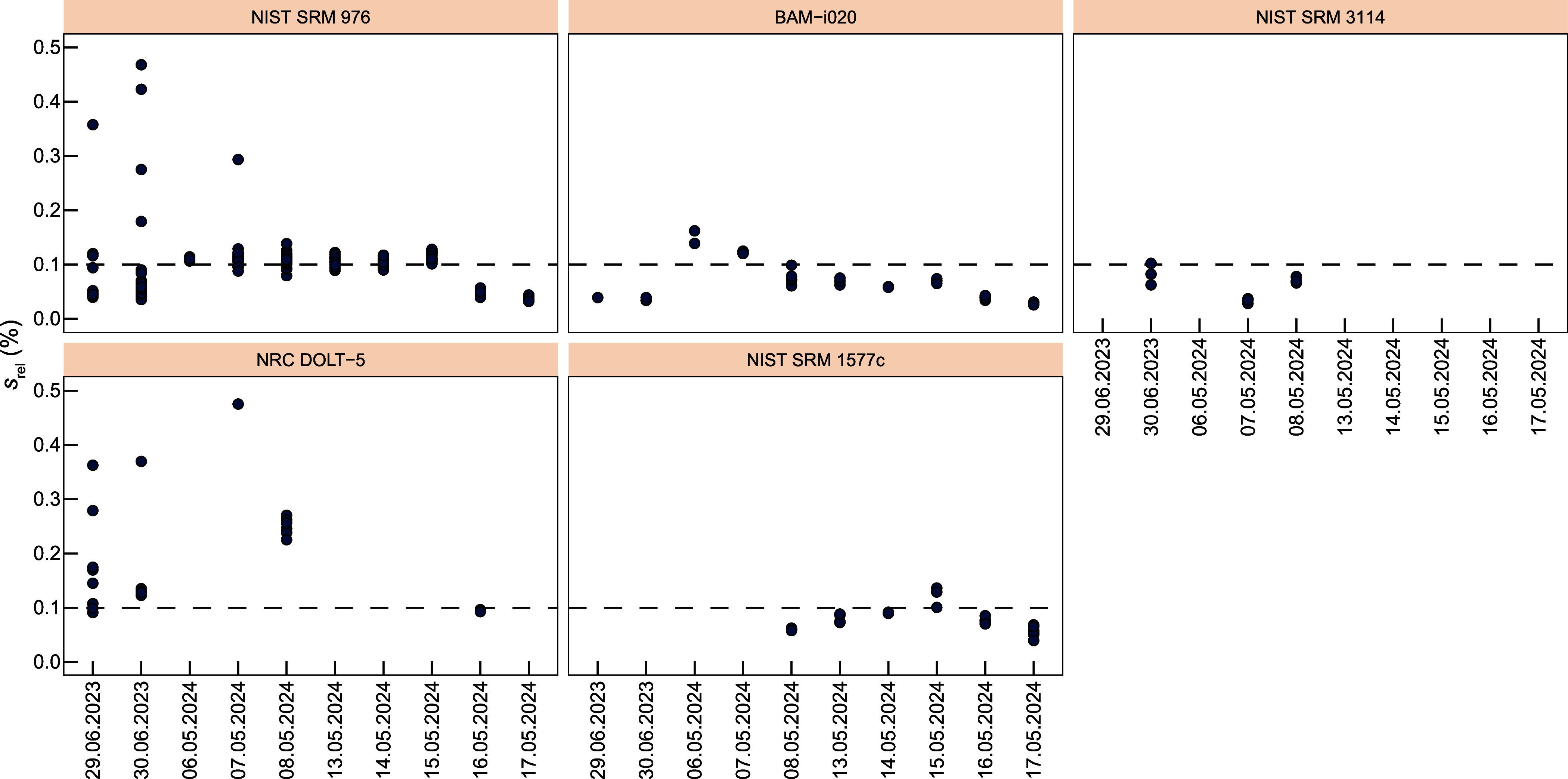
Relative standard deviation (*s*_rel_, in %) of ^65^Cu/^63^Cu ratios of the quality control and/or reference materials NIST SRM 976, BAM-i020, NIST SRM 3114, NRC DOLT-5, and NIST SRM 1577c prepared as gelatin droplets and measured during different measurement sessions. The dashed line depicts *s*_rel_ values of 0.1 %. Every data point depicts the *s*_rel_ values of 200 cycles within a single line scan.

Line scan measurements across the entire NIST SRM 976 gelatin standard were conducted using LA-MC-ICP-MS to determine if Cu diffusion resulted in the isotopic heterogeneity of the gelatin droplet. Figure 1C shows a constant measured *r*(^65^Cu/^63^Cu) ratio with a mean of 0.44534 and a relative standard deviation *s*_rel_ of 0.081% confirming a homogeneous isotope composition of the gelatin standard even in areas with changing Cu concentrations. The isotopic homogeneity of all of the prepared gelatin standard droplets (NIST SRM 976, BAM-i020, NIST SRM 3114, NRC DOLT-5, and NIST SRM 1577c) was evaluated by comparing the *s*_rel_ values for their raw ^65^Cu/^63^Cu ratio (Figure 2).

Throughout all analytical sessions, the different gelatin standards show an *s*_rel_ value of approximately ±0.1 %. Higher *s*_rel_ for some bracketing NIST SRM 976 measurements with values of up to 0.5% occurred after previous analysis of a high organic matrix material such as NCR DOLT-5 gelatin reference material or liver carcinoma samples indicating potential memory effects (Figure 2). Larger *s*_rel_ due to memory effects were limited by analyzing gelatin blanks (i.e., droplets of the pure fish skin gelatin solution) between the high organic matrix material and the following bracketing standard producing *s*_rel_ of ±0.1 %. Increased *s*_rel_ values were also observed for NRC DOLT-5 measurements, which are potentially related to a sodium argide interference discussed further below.

Gelatin standards produced in June 2023 were also tested during the analytical sessions of May 2024, as a bracketing standard and quality control reference materials, and results were indistinguishable from those with freshly produced gelatin droplets. Hence, the long-term stability of the gelatin standards is given over a period of a year.

### Inorganic Cu Isotope Reference Materials: NIST SRM 3114 and BAM-i020

**Figure 3:**
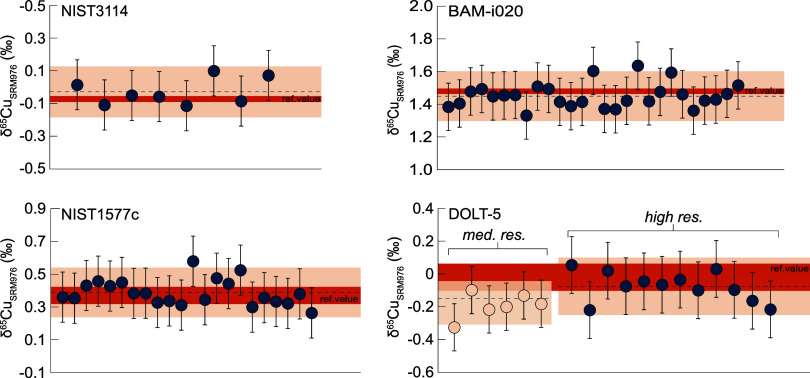
Results of LA-MC-ICP-MS analyses of inorganic and biological matrix Cu isotope reference materials. Note that medium and high mass resolution mode results are shown for NRC DOLT-5. The orange band depicts the solution reference value. The dashed line shows the mean value of LA-MC-ICP-MS data, and the light band shows the uncertainty of 2 *s*. Uncertainties of data points are given as 2 *s*. Reference values of the measured materials are displayed in Table 1.

The analytical routine was validated using inorganic Cu isotope standards NIST SRM 3114 and BAM-i020. Pure Cu solutions of these two standards were admixed with gelatin, and their dried droplets were analyzed against the bracketing standard NIST SRM 976. The resulting average isotope compositions of all gelatin reference materials analyzed by LA-MC-ICP-MS are summarized in Table 1. The precision of the data is given as intermediate precision (2 *s*), where the data for various droplets analyzed during different sessions is combined. The repeatabilities of delta values obtained from the same droplet analyzed in an individual sequence yield a smaller 2 *s* than the intermediate precision. Multiple LA-MC-ICP-MS analyses of NIST SRM 3114 gelatin droplets during several ablation sessions yielded an average Cu isotope composition of ‰ (*N* = 8) consistent within error with the published solution reference value of ‰ (Figure 3). Measurements of BAM-i020 gelatin droplets resulted in an average Cu isotope composition of ‰ (*N* = 27) similar to the published solution reference value of ‰ (Figure 3).

### Biological Matrix Reference Materials NIST SRM 1577c and NRC DOLT-5

Following the successful implementation of the method using inorganic reference materials, the LA-MC-ICP-MS routine was extended to reference materials with a biological matrix, specifically NIST SRM 1577c (bovine liver) and NRC DOLT-5 (dogfish liver). Similar to the inorganic reference materials, the dissolved biological matrix reference material admixed with gelatin was measured against the bracketing standard NIST SRM 976. Analyses of NIST SRM 1577c gelatin droplets during several ablation sessions gave an average Cu isotope composition of ‰ (*N* = 22) consistent with the published solution-based reference value of ‰^33^ (Figure 3).

Initial measurements of NRC DOLT-5 gelatin droplets resulted in an average Cu delta value of ‰ (*N* = 6), which is isotopically lighter than the published solution reference value of ‰^34^ (Figure 3). The initial analyses were performed in medium mass resolution mode, which was not capable of resolving the ^40^Ar^23^Na^+^ interference that is likely to arise due to the high Na/Cu ratio in NRC DOLT-5 compared to all other analyzed reference materials. The interference could be resolved by measuring in high resolution (Figure S2). Thus, subsequent measurements were performed in high-resolution mode resulting in an average Cu isotope composition of ‰ (*N* = 12) consistent with the published solution reference value (Figure 3).

The high mass resolution mode effectively mitigated the impact of the ^23^Na^40^Ar^+^ interference on ^63^Cu, ensuring reliable Cu isotope measurements in samples with a high Na content. Consequently, all samples with complex biological matrices, particularly with high Na/Cu ratios, were analyzed in high mass resolution mode to avoid spectral interference.

### Interference Induced by Na

Matrix elements can impact measured isotope ratios either by causing nonspectral mass fractionation effects or by inducing spectral interferences on Cu. Sodium has a high potential to generate the argide ^40^Ar^23^Na^+^ interfering with the ^63^Cu isobar, and this effect is exacerbated in biological samples due to their low Cu contents and high Na contents. High Na/Cu ratios were shown to shift Cu isotope compositions toward lighter isotope values as a result of the spectral interference in solution MC-ICP-MS.^35,36^

The same observation was made for the biological reference material DOLT-5, which possesses high Na/Cu ratios and yielded  values lighter than the reference value when analyzed by LA-MC-ICP-MS (Figure 3). Whereas ion-exchange chromatography prior to analysis is used for solution MC-ICP-MS to mitigate effects from matrix elements, LA-MC-ICP-MS relies on measuring in high-resolution mode, which yielded the correct  values for DOLT-5 (Figure 3). The disadvantage of high-resolution analysis is the loss of overall Cu intensities resulting in lower signal-to-noise ratios and, thus, higher relative standard deviations as well as larger uncertainty estimations, especially when analyzing samples with low Cu contents. Nonetheless, a high-resolution mode is required because of the high Na/Cu ratios of biological samples. Instruments with collision/reaction cell technologies (MC-ICP-MS/MS) are able to remove the ^40^Ar^23^Na^+^ interference by adding helium and destroying polyatomic ions through collisions with molecules,^37,38^ which would enable analysis in low resolution if coupled with laser ablation in the future.

### Application of *In Situ* Cu Isotope Analyses to In Vivo Rabbit VX2 Liver Tumor Samples

**Figure 4:**
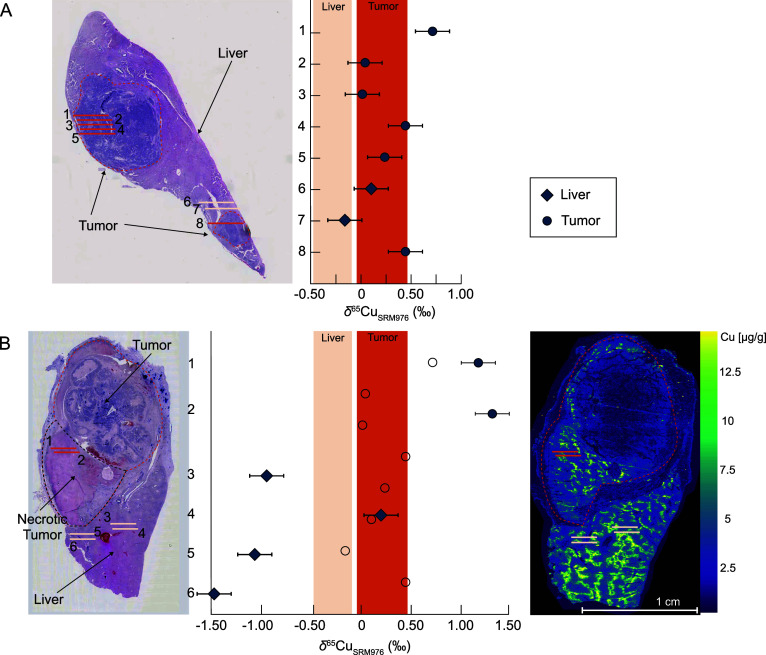
Copper isotope compositions of healthy and tumorous liver tissue in two VX2 liver tumor samples analyzed by LA-MC-ICP-MS. Laser ablation lines are shown on H&E staining images. Colored bars represent the range of Cu isotope variation for healthy human liver and hepatocellular carcinoma biopsies.^16^ (A) Delta values for liver and tumor of the control group animal. (B) Delta values for liver and tumor of the microwave ablation treated group sample. A Cu elemental content image measured on a parallel tissue slide by LA-ICP-ToF-MS is shown, as well. Empty circles show the data of the control group sample in panel A.

The developed LA-MC-ICP-MS routine was used to analyze two rabbit liver samples aiming to distinguish between the Cu isotope composition of healthy and tumorous areas. Parallel tissue sections with H&E staining were used as guidance to ablate tumor areas and healthy parts of the liver. In the control group sample  values ranged from −0.2 ‰ to 0.7 ‰ (Figure 4A). Laser ablation lines on healthy areas yielded  values from −0.2 ‰ to 0.1 ‰, whereas analyses on tumorous tissue gave slightly heavier  values ranging from 0.0 ‰ to 0.7 ‰ (Figure 4A).

For the microwave ablation group (MWA-group) sample, an additional parallel section was analyzed with LA-ICP-ToF-MS to supply an image of the elemental Cu distribution (Figure 4B). Therefore, the position of laser ablation lines could be selected based on regions of higher Cu content enabling larger signal intensities in addition to the information from H&E staining. The  values of the MWA-group sample ranged from −1.5 ‰ to 1.3 ‰ (Figure 4B). Measurements of healthy tissue resulted in  values ranging from −1.5 ‰ to 0.2 ‰, whereas analyses on tumor areas yielded heavier  values from 1.2 ‰ to 1.3 ‰ (Figure 4B).

Results of both samples reveal an isotopic distinction between healthy and tumorous as well as necrotic tissues. Tumor and necrotic samples are generally in the positive delta area, whereas healthy liver samples are in the negative delta range. The isotopic difference between the two tissue areas is more distinct in the MWA-group sample, where the information on both H&E staining and the ToF image was crucial in selecting the laser ablation areas.

### Isotope Fractionation of Hepatocellular Carcinoma

The liver is a major storage site for Cu and plays an essential role in Cu homeostasis. Changes in metabolic processes in the liver, such as oxidative phosphorylation, hypoxia, and angiogenesis, have been linked to isotope fractionation.^3^ In situ Cu isotope measurements of VX2 tumor and necrotic tissue revealed heavier  values relative to the adjacent healthy liver tissue (Figure 4). Stronger Cu isotopic variations were evident in the microwave ablation group sample, where analyzed areas were selected according to the elemental Cu concentration images of parallel tissue sections. Discerning if the larger variation is due to the improved choice of ablation area via Cu ToF images or related to microwave treatment relative to the control group will be revealed in future work on larger cohorts.

Nonetheless, the observed isotopic differences are consistent with Cu isotope data of HCC as well as ovarian cancer biopsies that showed enrichment of ^65^Cu relative to surrounding healthy tissue and concomitant isotopically lighter blood serum.^16,18^ Studies of HepG2 cells, an in vitro model of human hepatocytes, revealed that oxidative stress causes intracellular enrichment of ^65^Cu, whereas ^63^Cu is preferentially excreted through membrane transporters.^39,40^ Combined biopsy, serum, and cell model results therefore suggest that increased lactate levels in the hypoxic tumor environment result in elevated carboxyl groups with a bonding environment that favors ^65^Cu leading to enriched  compositions of cancer cells.

To fully understand the response of cellular Cu isotope compositions to oxidative stress and disease progression, a combination of *in vitro* and *in vivo* studies is paramount.

### Uncertainty Estimation for  Values of Gelatin Reference Materials

The combined uncertainty estimation for the calculated  values is based on previous approaches.^41^ The budget includes contributions from the isotope ratio measurements of sample and standards (*R*), gelatin blank (κ1), a heterogeneity component (κ2), and a matrix effect component (κ3). Estimated uncertainties for the gelatin-based reference materials range between 0.46 ‰ and 0.54 ‰. Details are listed in Table 1.

The uncertainty budget is predominantly governed by the gelatin blank contribution, which can potentially be decreased by using other materials such as agarose gel in further studies. Agarose gel is used in bioimaging, akin to gelatin standards,^42^ and could carry a lower elemental matrix. In addition, the application of 10^13^ Ω Faraday cup amplifiers would allow for better signal-to-noise ratios,^43^ and therefore reduce uncertainty estimations. Uncertainties of biomedical sample data could be decreased by calculating Δ values (*i.e*., the difference between the isotope deltas of two substances, here liver and tumor), which negates various contributions to the uncertainty estimation. The isotopic difference, Δ, is given by eq 2:
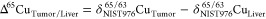
2

The calculated  for the microwave ablation group are on the order of two delta units, and hence, resolvable with the estimated uncertainties and showing a clear isotopic difference between the two types of tissues.

## Conclusions

We developed an analytical routine for *in situ* measurements of Cu isotope variations of biomedical samples using LA-MC-ICP-MS. Matrix-matched gelatin-based Cu isotope standards were produced to correct for instrumental sources of isotope fractionation. Analyses of reference materials admixed with gelatin yielded accurate and precise  values consistent with certified values advocating for measurements of biomedical samples. To test the method, VX2 tumor cells in rabbit livers, a model system for human hepatocellular carcinoma (HCC) were analyzed and showed distinct Cu isotope variations between tumor and healthy tissues agreeing with previous work on HCC biopsies analyzed with solution-based MC-ICP-MS. The novel method will substantially boost the field of isotope metallomics as it allows spatial resolution of isotopic variations and faster sample throughput. A current shortcoming is the need to analyze in high-resolution mode to avoid spectral interferences, which could be evaded when combined with new collision/reaction cell MC-ICP-MS machines permitting the analysis of even smaller sample amounts.

## Supporting Information Available

Details on instrumental set up of laser ablation MC-ICP-MS and instrumental mass fractionation correction, detailed description of LA-ICP-ToF-MS elemental imaging routine, and Supporting Figures S1 and S2 and Tables S1 and S2 (PDF)
