# Supplementary material for: Advancing Biomarker Research: In Situ Cu Isotope Analysis in Liver Tumors by LA-MC-ICP-MS
Source: Anal Chem. 2025 Feb 18;97(8):4425–32. doi: 10.1021/acs.analchem.4c05626 (PMC11883731; doi:10.1021/acs.analchem.4c05626)
Supplement: Supplementary file 1 — ac4c05626_si_001.pdf [file ac4c05626_si_001.pdf]

Supporting Information

Advancing biomarker research: In situ Cu isotope analysis in liver tumors by LA-MC-ICP-MS

Mathias Schannor,*,† Marcus Oelze,† Heike Traub,† Yubei He,‡ Robin Schmidt,‡ Luisa Heidemann,‡ Lynn Jeanette Savic,‡ Jochen Vogl,† and Björn Meermann†

†*Bundesanstalt für Materialforschung und -prüfung (BAM), Richard-Willstätter-Straße 11, 12489 Berlin, Germany.*

‡*Department of Radiology, Charité - Universitätsmedizin Berlin Campus Virchow Klinikum (CVK), Augustenburger Platz 1, 13353 Berlin, Germany.*

E-mail: mathias.schannor@bam.de

Laser Ablation MC-ICP-MS

Intrumental details are listed in Table S1 A combination of standard-sample-bracketing (SSB) and internal normalisation using Ni as internal standard was employed to correct Cu isotope ratios for instrumental mass bias. The Ni solution was aspirated using an Apex Q (Elemental Scientific Inc, Omaha, USA) desolvating nebulizer. The correction is based on the assumption that instrumental mass bias for Ni behaves similarly to that of Cu within a session. Compared to previous studies[1,2] Ni correction was preferred over Ga because of $^{40}Ar^{31}P^{+}$ isobaric interferences on ^{71}Ga caused by large contents of phosphorous in biological samples. Initially, the instrumental mass fractionation factor for Ni (f_{Ni}), which is assumed to approximate the unknown Cu instrumental mass fractionation factor (f_{Cu}), is determined

from simultaneous measurements of Ni isotope ratios for each individual measurement, as described by equation 1.

$$f_{Ni} = \frac{ln(R^*(^{61}\text{Ni}/^{60}\text{Ni})/r(^{61}\text{Ni}/^{60}\text{Ni})}{ln(A_r(^{61}\text{Ni})/A_r(^{60}\text{Ni})} \quad (1)$$

Where R^* is the *"true"* Ni isotope ratio of the Ni solution used to correct the mass bias, $r(^{61}\text{Ni}/^{60}\text{Ni})$ is the measured Ni isotope ratio, and A_r the relative atomic masses of the two measured Ni isotopes (Note: knowledge of the *true* isotope ratio, R, is not necessary when applying standard sample bracketing; here, we use the IUPAC value for $R^*(^{61}\text{Ni}/^{60}\text{Ni})$ = 0.0435)[3]). Then, in the absence of a known f_{Cu} , the f_{Ni} is used to correct the measured Cu isotope ratio following equation 2:

$$R^*(^{65}\text{Cu}/^{63}\text{Cu}) = r(^{65}\text{Cu}/^{63}\text{Cu}) \times A_r(^{65}\text{Cu})/A_r(^{63}\text{Cu})^{f_{\text{Ni}}} \quad (2)$$

However, equation 2 cannot be used to directly calculate absolute $R(^{65}\text{Cu}/^{63}\text{Cu})$ isotope ratios because the actual isotope ratio of the Ni doping solution is unknown. Furthermore, f_{Cu}=f_{Ni} is an approximation and the ratio of the two exponents f_{Cu}/f_{Ni} is not constant.[4] Thus the conversion to delta values relative to the bracketing standard NIST SRM 976 is done by normalizing the Ni-corrected Cu isotope ratios relative to the mean Cu isotope value of NIST SRM 976 measured before and after each sample, following equation 3:

$$\delta^{65/63}_{\text{NIST 976}}(\text{Cu}) = \left(\frac{R^*(^{65}\text{Cu}/^{63}\text{Cu})_{\text{sample}}}{R^*(^{65}\text{Cu}/^{63}\text{Cu})_{\text{NIST SRM 976}}} - 1 \right) \quad (3)$$

We report Cu isotope values in the delta notation according to Coplen[5] as $\delta^{65/63}_{\text{NIST 976}}(\text{Cu})$ relative to the international isotope measurement standard NIST SRM 976 in per mil (‰).

The in-house prepared gelatin material spiked with NIST SRM 976 as Cu source served as the bracketing material. Furthermore, in each measurement session, a set of reference materials, such as DOLT-5 and NIST SRM 1577c, mixed with gelatin and deposited on glass slides, were regularly analyzed between sample measurements.

To process the raw isotope data and correct for background effects, we followed a previously published protocol.[6] This protocol involved applying specific data acceptance and rejection criteria. Notably, only measured r(^{65}Cu/^{63}Cu) ratios deviating by less than 3 standard deviations from the sample mean (criterion 'a') were considered for calculation. Additionally, results with a mass bias drift of less than 0.30 ‰ between the two bracketing calibrators (criterion 'b') were accepted and reported in this study.

Table S1: Laser instrument settings used for the LA-MC-ICP-MS Cu isotope ratio measurement on the Thermo Neptune Plus MC-ICP-MS.

NWR213 LA system settings:	
He transport gas flow / (mL/min)	850
Beam size / (μm)	110
Mask shape	circle
Repetition rate / (Hz)	20
Scan velocity / (μm/s)	10
Fluence / (J/cm^2)	0.5
Wavelength / (nm)	213

LA-ICP-ToF-MS imaging

Machine details are listed in Table S2 A square laser beam of 20 µm × 20 µm was used to produce high resolution chemical maps. The samples were scanned underneath a pulsed beam with a repetition rate of 100 Hz and a fluence of 10 J/cm^2 and ablation spots were lined up edge-to-edge across each line and between consecutive lines without overlapping areas. Laser ablation in the TwoVol3 ablation chamber was carried out in a helium atmosphere using a combined helium flow of 0.65 L min^{-1} and an additional argon flow of 0.9 L min^{-1} injected at the torch of the ICP-ToF-MS. The injection of H_2 and He into the collision/reaction cell (CRC) of the ICP-ToF-MS reduces $^{40}Ar^+$ and $^{40}Ar^{40}Ar^+$ interferences.[7] A H_2/He gas (8.1 % H_2 and 91.9 % He) was used with a flow rate of approximately 4.0 mL min^{-1}. Instrumental settings were optimised tuning with NIST SRM610 glass focussing on high intensities, low

oxide formation rates and low elemental fractionation based on $^{238}U^+/^{232}Th^+$ values close to 1. The effective wash-out time was 10 ms (full-width at 1 percent maximum, FW0.01M) based on the signal from ^{28}Si of individual pulses on NIST SRM 610 glass and from the ^{63}Cu signal of individual pulses on gelatin standards.

Data acquisition was triggered for every ablation position using the laser ablation workflow of TofPilot (Tofwerk AG, Thun, Switzerland) acquiring the entire image as a single Hierachical Data Format (HDF5) file. Gas blank signals were measured before each sample and calibration material, respectively. Data post-processing of the acquired images and reference materials was conducted in two steps. The raw data were first processed with the Tofware software (TOFWERK AG, Thun, Switzerland) to select isotopes, refine peak shapes, perform mass calibration, and model and subtract baseline signal intensities. Afterwards, the modified HDF5 files were loaded in Iolite v4 software (Elemental Scientific Laser, Bozeman, USA) and the data were reduced using the "3D Trace Elements" (3D-TE-DRS) data reduction scheme.[8–10] Gelatin multi-element standards (BIO-logi-CAL standard 2, 48 elements; aemas, Oosterhout, The Netherlands) were used as calibration material following recently established procedures[11] and analysed before and after samples.

Table S2: Instrument settings used for LA-ICP-ToF-MS imaging.

icpTOF 2R settings:	
RF Power / (W)	1550
Ar cool gas flow / (L/min)	14
Ar auxiliary gas flow/ (L/min)	0.8
Ar nebuliser gas flow/ (L/min)	0.95
Sampling depth / (mm)	4
CRC H_2/He gas flow / (mL/min)	4.0
NWRimage LA system settings:	
He chamber gas flow / (mL/min)	300
He cup gas flow / (mL/min)	350
Beam size / (μm)	20
Mask shape	square
Repetition rate / (Hz)	100
scan velocity / (μm/s)	2000
Fluence / (J/cm^2)	10
Wavelength / (nm)	266

Supporting Figures

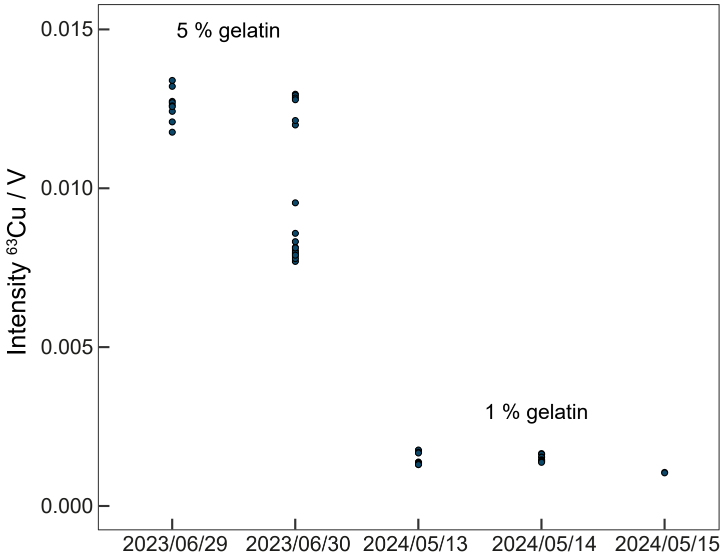

Figure S1: Measured intensity of the ^{63}Cu signal of different gelatin blank droplets prepared with gelatin mass fractions of 1 % and 5 %, respectively.

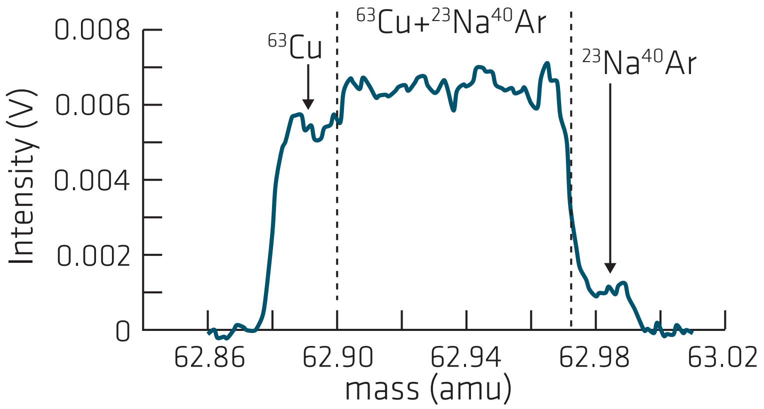

Figure S2: Mass scan of blank solution with 500 ng/g Na.

References

(1) Hou, Q.; Zhou, L.; Gao, S.; Zhang, T.; Feng, L.; Yang, L. Use of Ga for mass bias correction for the accurate determination of copper isotope ratio in the NIST SRM

3114 Cu standard and geological samples by MC-ICPMS. *Journal of Analytical Atomic Spectrometry* **2016**, *31*, 280–287.

(2) Sullivan, K.; Layton-Matthews, D.; Leybourne, M.; Kidder, J.; Mester, Z.; Yang, L. Copper isotopic analysis in geological and biological reference materials by MC-ICP-MS. *Geostandards and Geoanalytical Research* **2020**, *44*, 349–362.

(3) Meija, J.; Coplen, T. B.; Berglund, M.; Brand, W. A.; De Bièvre, P.; Gröning, M.; Holden, N. E.; Irrgeher, J.; Loss, R. D.; Walczyk, T.; others Isotopic compositions of the elements 2013 (IUPAC Technical Report). *Pure and Applied Chemistry* **2016**, *88*, 293–306.

(4) Maréchal, C. N.; Télouk, P.; Albarède, F. Precise analysis of copper and zinc isotopic compositions by plasma-source mass spectrometry. *Chemical geology* **1999**, *156*, 251–273.

(5) Coplen, T. B. Guidelines and recommended terms for expression of stable-isotope-ratio and gas-ratio measurement results. *Rapid communications in mass spectrometry* **2011**, *25*, 2538–2560.

(6) Schuessler, J. A.; von Blanckenburg, F. Testing the limits of micro-scale analyses of Si stable isotopes by femtosecond laser ablation multicollector inductively coupled plasma mass spectrometry with application to rock weathering. *Spectrochimica Acta Part B: Atomic Spectroscopy* **2014**, *98*, 1–18.

(7) Burger, M.; Hendriks, L.; Kaeslin, J.; Gundlach-Graham, A.; Hattendorf, B.; Günther, D. Characterization of inductively coupled plasma time-of-flight mass spectrometry in combination with collision/reaction cell technology–insights from highly time-resolved measurements. *Journal of Analytical Atomic Spectrometry* **2019**, *34*, 135–146.

(8) Paton, C.; Hellstrom, J.; Paul, B.; Woodhead, J.; Hergt, J. Iolite: Freeware for the visualisation and processing of mass spectrometric data. *Journal of Analytical Atomic Spectrometry* **2011**, *26*, 2508–2518.

(9) Paul, B.; Petrus, J.; Savard, D.; Woodhead, J.; Hergt, J.; Greig, A.; Paton, C.; Rayner, P. Time resolved trace element calibration strategies for LA-ICP-MS. *Journal of Analytical Atomic Spectrometry* **2023**, *38*, 1995–2006.

(10) Savard, D.; Dare, S.; Bédard, L. P.; Barnes, S.-J. A New Mapping Protocol for Laser Ablation (with Fast-Funnel) Coupled to a Time-of-Flight Mass Spectrometer (LA-FF-ICP-ToF-MS) for the Rapid, Simultaneous Quantification of Multiple Minerals. *Geostandards and Geoanalytical Research* **2023**, *47*, 243–265.

(11) Schweikert, A.; Theiner, S.; Wernitznig, D.; Schoeberl, A.; Schaier, M.; Neumayer, S.; Keppler, B. K.; Koellensperger, G. Micro-droplet-based calibration for quantitative elemental bioimaging by LA-ICPMS. *Analytical and Bioanalytical Chemistry* **2022**, *414*, 485–495.
